# Supplementary material for: Modeling glioblastoma heterogeneity as a dynamic network of cell states
Source: Mol Syst Biol. 2021 Sep 16;17(9):e10105. doi: 10.15252/msb.202010105 (PMC8444284; doi:10.15252/msb.202010105)
Supplement: Supplementary file 5 — Source Data for Figure 3 [file MSB-17-e10105-s001.zip › Figure3A_sourcedata/GSEA_3065/nosigdb_state2.GseaPreranked.1622718419215/NB_KI_CLUSTER17.html]

Details for gene set NB\_KI\_CLUSTER17[GSEA]

|  || Dataset | state2 |
| Phenotype | NoPhenotypeAvailable |
| Upregulated in class | na\_neg |
| GeneSet | NB\_KI\_CLUSTER17 |
| Enrichment Score (ES) | -0.64310694 |
| Normalized Enrichment Score (NES) | -2.5933182 |
| Nominal p-value | 0.0 |
| FDR q-value | 0.0 |
| FWER p-Value | 0.0 |
Table: GSEA Results Summary

  

Fig 1: Enrichment plot: NB\_KI\_CLUSTER17      
 Profile of the Running ES Score & Positions of GeneSet Members on the Rank Ordered List

  

| PROBE | GENE SYMBOL | GENE\_TITLE | RANK IN GENE LIST | RANK METRIC SCORE | RUNNING ES | CORE ENRICHMENT || 1 | NFIC |  |  | 543 | 0.174 | -0.0456 | No |
| 2 | TUBB6 |  |  | 622 | 0.160 | -0.0422 | No |
| 3 | GNG11 |  |  | 698 | 0.151 | -0.0392 | No |
| 4 | EMP2 |  |  | 826 | 0.135 | -0.0429 | No |
| 5 | EPB41L2 |  |  | 1200 | 0.105 | -0.0753 | No |
| 6 | SPRY1 |  |  | 1623 | 0.081 | -0.1147 | No |
| 7 | CSTB |  |  | 1638 | 0.080 | -0.1103 | No |
| 8 | EGR1 |  |  | 1691 | 0.077 | -0.1102 | No |
| 9 | JUN |  |  | 1805 | 0.072 | -0.1170 | No |
| 10 | PLSCR1 |  |  | 1853 | 0.070 | -0.1169 | No |
| 11 | RCAN1 |  |  | 1903 | 0.068 | -0.1172 | No |
| 12 | REXO2 |  |  | 1935 | 0.067 | -0.1156 | No |
| 13 | FTH1 |  |  | 2254 | 0.055 | -0.1457 | No |
| 14 | RHOC |  |  | 2261 | 0.055 | -0.1423 | No |
| 15 | CNN3 |  |  | 2349 | 0.052 | -0.1478 | No |
| 16 | MT1X |  |  | 2538 | 0.046 | -0.1646 | No |
| 17 | RHOB |  |  | 2690 | 0.042 | -0.1777 | No |
| 18 | NFIA |  |  | 2796 | 0.039 | -0.1861 | No |
| 19 | ANXA5 |  |  | 2812 | 0.039 | -0.1849 | No |
| 20 | SOD2 |  |  | 3366 | 0.026 | -0.2424 | No |
| 21 | H2AFJ |  |  | 3640 | 0.022 | -0.2701 | No |
| 22 | PDLIM2 |  |  | 3807 | 0.019 | -0.2866 | No |
| 23 | TNFSF12 |  |  | 3926 | 0.017 | -0.2980 | No |
| 24 | RAB13 |  |  | 3928 | 0.017 | -0.2969 | No |
| 25 | TSPAN4 |  |  | 3929 | 0.017 | -0.2957 | No |
| 26 | NDRG2 |  |  | 4043 | 0.015 | -0.3067 | No |
| 27 | LAMA4 |  |  | 4068 | 0.014 | -0.3082 | No |
| 28 | SPRED1 |  |  | 4165 | 0.013 | -0.3176 | No |
| 29 | PHACTR2 |  |  | 4339 | 0.010 | -0.3355 | No |
| 30 | BDH2 |  |  | 4538 | 0.007 | -0.3562 | No |
| 31 | CLEC11A |  |  | 4738 | 0.004 | -0.3773 | No |
| 32 | CLIC4 |  |  | 4778 | 0.003 | -0.3813 | No |
| 33 | IL1RAP |  |  | 4887 | 0.001 | -0.3928 | No |
| 34 | TRIP10 |  |  | 4955 | 0.000 | -0.4000 | No |
| 35 | PLA2G16 |  |  | 5038 | -0.001 | -0.4087 | No |
| 36 | GULP1 |  |  | 5158 | -0.003 | -0.4213 | No |
| 37 | TAX1BP3 |  |  | 5161 | -0.003 | -0.4213 | No |
| 38 | DNAJB4 |  |  | 5172 | -0.003 | -0.4222 | No |
| 39 | PLXND1 |  |  | 5560 | -0.009 | -0.4631 | No |
| 40 | PLEKHA4 |  |  | 5925 | -0.015 | -0.5011 | No |
| 41 | CCDC80 |  |  | 6077 | -0.018 | -0.5160 | No |
| 42 | GPX8 |  |  | 6123 | -0.019 | -0.5194 | No |
| 43 | HES1 |  |  | 6191 | -0.020 | -0.5252 | No |
| 44 | ERRFI1 |  |  | 6445 | -0.024 | -0.5506 | No |
| 45 | FAM126A |  |  | 6580 | -0.027 | -0.5630 | No |
| 46 | LTBP4 |  |  | 6665 | -0.029 | -0.5699 | No |
| 47 | GRAMD2B |  |  | 6703 | -0.030 | -0.5717 | No |
| 48 | HEG1 |  |  | 6782 | -0.031 | -0.5778 | No |
| 49 | BLVRB |  |  | 7000 | -0.036 | -0.5984 | No |
| 50 | CTSO |  |  | 7105 | -0.039 | -0.6067 | No |
| 51 | RPS27L |  |  | 7139 | -0.040 | -0.6073 | No |
| 52 | AEBP1 |  |  | 7162 | -0.041 | -0.6066 | No |
| 53 | ITPRIPL2 |  |  | 7268 | -0.044 | -0.6147 | No |
| 54 | NBL1 |  |  | 7312 | -0.045 | -0.6160 | No |
| 55 | SPATS2L |  |  | 7352 | -0.046 | -0.6168 | No |
| 56 | RGL1 |  |  | 7385 | -0.047 | -0.6168 | No |
| 57 | HSPG2 |  |  | 7413 | -0.048 | -0.6162 | No |
| 58 | QKI |  |  | 7478 | -0.050 | -0.6194 | No |
| 59 | RUNX1 |  |  | 7507 | -0.051 | -0.6187 | No |
| 60 | PHLDA3 |  |  | 7590 | -0.053 | -0.6236 | No |
| 61 | FTL |  |  | 7693 | -0.056 | -0.6304 | No |
| 62 | IL13RA1 |  |  | 7762 | -0.058 | -0.6335 | No |
| 63 | TRIP6 |  |  | 7846 | -0.061 | -0.6379 | No |
| 64 | RASSF4 |  |  | 7851 | -0.061 | -0.6338 | No |
| 65 | LOXL2 |  |  | 7871 | -0.062 | -0.6313 | No |
| 66 | PLPP3 |  |  | 7911 | -0.063 | -0.6308 | No |
| 67 | TNFRSF12A |  |  | 7931 | -0.064 | -0.6282 | No |
| 68 | BACE2 |  |  | 8071 | -0.070 | -0.6380 | Yes |
| 69 | IL17RC |  |  | 8076 | -0.070 | -0.6333 | Yes |
| 70 | MATN2 |  |  | 8102 | -0.071 | -0.6307 | Yes |
| 71 | BAG3 |  |  | 8104 | -0.071 | -0.6256 | Yes |
| 72 | FBN1 |  |  | 8107 | -0.072 | -0.6205 | Yes |
| 73 | UGCG |  |  | 8171 | -0.076 | -0.6217 | Yes |
| 74 | ANXA4 |  |  | 8188 | -0.077 | -0.6178 | Yes |
| 75 | ERBB3 |  |  | 8230 | -0.079 | -0.6165 | Yes |
| 76 | TNS3 |  |  | 8231 | -0.079 | -0.6107 | Yes |
| 77 | IFI6 |  |  | 8257 | -0.080 | -0.6075 | Yes |
| 78 | NPC2 |  |  | 8297 | -0.083 | -0.6056 | Yes |
| 79 | TRAM1 |  |  | 8333 | -0.085 | -0.6031 | Yes |
| 80 | CREB3L2 |  |  | 8341 | -0.085 | -0.5977 | Yes |
| 81 | NOTCH2 |  |  | 8358 | -0.086 | -0.5931 | Yes |
| 82 | WIPI1 |  |  | 8505 | -0.096 | -0.6017 | Yes |
| 83 | AHR |  |  | 8513 | -0.097 | -0.5953 | Yes |
| 84 | NECTIN2 |  |  | 8580 | -0.102 | -0.5950 | Yes |
| 85 | PCOLCE |  |  | 8685 | -0.110 | -0.5980 | Yes |
| 86 | LRP10 |  |  | 8697 | -0.111 | -0.5911 | Yes |
| 87 | WWTR1 |  |  | 8752 | -0.117 | -0.5883 | Yes |
| 88 | ZEB2 |  |  | 8786 | -0.120 | -0.5831 | Yes |
| 89 | PLK2 |  |  | 8803 | -0.123 | -0.5758 | Yes |
| 90 | PLPP1 |  |  | 8821 | -0.125 | -0.5684 | Yes |
| 91 | MYDGF |  |  | 8844 | -0.128 | -0.5614 | Yes |
| 92 | S100A13 |  |  | 8880 | -0.133 | -0.5554 | Yes |
| 93 | ANXA2 |  |  | 8891 | -0.134 | -0.5466 | Yes |
| 94 | FNDC3B |  |  | 8902 | -0.136 | -0.5377 | Yes |
| 95 | TGFBR2 |  |  | 8916 | -0.137 | -0.5291 | Yes |
| 96 | RCN1 |  |  | 8929 | -0.138 | -0.5202 | Yes |
| 97 | SVIL |  |  | 8981 | -0.146 | -0.5150 | Yes |
| 98 | PON2 |  |  | 9030 | -0.154 | -0.5088 | Yes |
| 99 | FGFR1 |  |  | 9034 | -0.155 | -0.4978 | Yes |
| 100 | PRNP |  |  | 9068 | -0.161 | -0.4895 | Yes |
| 101 | IL6ST |  |  | 9090 | -0.164 | -0.4797 | Yes |
| 102 | SPTBN1 |  |  | 9111 | -0.169 | -0.4695 | Yes |
| 103 | PLAT |  |  | 9145 | -0.176 | -0.4601 | Yes |
| 104 | EMP1 |  |  | 9156 | -0.179 | -0.4481 | Yes |
| 105 | AHNAK |  |  | 9183 | -0.185 | -0.4372 | Yes |
| 106 | GOLIM4 |  |  | 9189 | -0.187 | -0.4241 | Yes |
| 107 | DAG1 |  |  | 9192 | -0.187 | -0.4106 | Yes |
| 108 | GRN |  |  | 9251 | -0.214 | -0.4011 | Yes |
| 109 | CTSB |  |  | 9255 | -0.215 | -0.3857 | Yes |
| 110 | CALU |  |  | 9278 | -0.223 | -0.3716 | Yes |
| 111 | LAMC1 |  |  | 9286 | -0.226 | -0.3557 | Yes |
| 112 | GPNMB |  |  | 9290 | -0.228 | -0.3393 | Yes |
| 113 | CALR |  |  | 9310 | -0.237 | -0.3240 | Yes |
| 114 | PMEPA1 |  |  | 9313 | -0.241 | -0.3065 | Yes |
| 115 | CTSD |  |  | 9320 | -0.246 | -0.2891 | Yes |
| 116 | LRP1 |  |  | 9338 | -0.259 | -0.2719 | Yes |
| 117 | ITGAV |  |  | 9346 | -0.269 | -0.2529 | Yes |
| 118 | PMP22 |  |  | 9347 | -0.271 | -0.2330 | Yes |
| 119 | CD81 |  |  | 9356 | -0.281 | -0.2132 | Yes |
| 120 | CD59 |  |  | 9358 | -0.283 | -0.1925 | Yes |
| 121 | PSAP |  |  | 9363 | -0.294 | -0.1714 | Yes |
| 122 | LAPTM4A |  |  | 9368 | -0.299 | -0.1499 | Yes |
| 123 | EDNRB |  |  | 9369 | -0.301 | -0.1279 | Yes |
| 124 | LAMP2 |  |  | 9380 | -0.322 | -0.1053 | Yes |
| 125 | GPM6B |  |  | 9389 | -0.344 | -0.0809 | Yes |
| 126 | MIR99AHG |  |  | 9395 | -0.354 | -0.0555 | Yes |
| 127 | ITM2B |  |  | 9399 | -0.371 | -0.0286 | Yes |
| 128 | VMP1 |  |  | 9417 | -0.448 | 0.0025 | Yes |
Table: GSEA details [plain text format]

  

Fig 2: NB\_KI\_CLUSTER17: Random ES distribution      
 Gene set null distribution of ES for **NB\_KI\_CLUSTER17**
